# miR-20a regulates expression of the iron exporter ferroportin in lung cancer

**DOI:** 10.1007/s00109-015-1362-3

**Published:** 2015-11-12

**Authors:** Kamesh R. Babu, Martina U. Muckenthaler

**Affiliations:** Department of Pediatric Hematology, Oncology, and Immunology, University of Heidelberg, Heidelberg, Germany; Molecular Medicine Partnership Unit, University of Heidelberg, Heidelberg, Germany

**Keywords:** Ferroportin, miR-20a, Lung cancer, Proliferation, Colony formation

## Abstract

**Abstract:**

Ferroportin (FPN) exports iron from duodenal enterocytes, macrophages, and hepatocytes to maintain systemic iron homeostasis. In addition, FPN is expressed in various cancer cells. Here, we show that in lung cancer, FPN expression is regulated by miR-20a. Within the FPN-3′-untranslated region (3′UTR), we identify and experimentally validate three evolutionarily conserved target sites for the microRNA (miRNA) members of the miR-17 seed family, including miR-20a. Our analysis of RNA sequencing data from patients with lung adenocarcinoma (LUAD) and lung squamous cell carcinoma (LUSC) revealed that FPN messenger RNA (mRNA) levels are significantly decreased in tumor compared to matched healthy tissue, while miR-20a levels are increased. A significant negative correlation of miR-20a and FPN expression was observed. Functional studies further demonstrate that FPN is post-transcriptionally regulated by miR-20a in non-small cell lung cancer (NSCLC) cells and that overexpression or knockdown of miR-20a or FPN affects NSCLC proliferation and colony formation. Taken together, our data suggest that increased expression of miR-20 in lung cancer may decrease iron export, leading to intracellular iron retention, which, in turn, favors cell proliferation.

**Key messages:**

- miR-20a controls expression of the iron exporter ferroportin (FPN) by binding to highly conserved target sites in its 3′UTR.
- Expression of miR-20a is inversely correlated to FPN in lung cancer.
- Low FPN expression stimulates proliferation and colony formation of non-small cell lung cancer (NSCLC) cells, possibly by increasing iron availability for cancer cell proliferation.

**Electronic supplementary material:**

The online version of this article (doi:10.1007/s00109-015-1362-3) contains supplementary material, which is available to authorized users.

## Introduction

MicroRNAs (miRNAs) are a class of small non-coding RNAs that negatively regulate gene expression by base pairing to partially complementary sites within the 3′-untranslated regions (3′UTR) of target messenger RNAs (mRNA) [1]. Binding of miRNA to its target mRNA causes translation repression [2] and/or mRNA degradation [3]. Genome-wide profiling of miRNA expression in cancer samples and their corresponding normal tissues showed a distinguished signature between tumoral and normal cells [4, 5], which is often associated with cancer prognosis and progression [6–8]. In particular, a well-characterized polycistronic miRNA cluster, the miR-17-92 family, accomplishes pleiotrophic functions in cancer [9, 10]. It is highly conserved among vertebrates [11] and has two mammalian paralogs: the miR-106a-363 and the miR-106b-25 cluster [12]. The miRNAs encoded by miR-17-92 and its two paralogs can be categorized into four “seed” families according to their seed sequences: miR-17 family (including miR-17, miR-20a, miR-20b, miR-106a, miR-106b and miR-93), miR-18 family (miR-18a and miR-18b), miR-19 family (miR-19a, miR-19b-1 and miR-19b-2), and miR-92 family (miR-92a-1, miR-92a-2, miR-25, and miR-363) [13]. Overexpression of the miR-17-92 cluster has been observed in a variety of hematological malignancies and solid tumors including B cell lymphoma, B cell chronic lymphocytic leukemia (CLL), retinoblastoma, meduloblastoma, osteosarcoma, and cancers of the colon, pancreas, breast, lung, kidney, and liver (as reviewed in [13]). The miRNA members of the miR-17-92 cluster contribute to cancer pathogenesis by directly regulating genes associated with cell growth and proliferation. The miR-17 family (miR-17 and miR-20a) triggers rapid proliferation by repressing the cell cycle inhibitors p21 (CDKN1A) and p57 (CDKN1C) [14, 15] as well as by directly targeting the proapoptotic protein Bim (BCL211) [16] and the tumor suppressor gene phosphatase and tensin homolg (PTEN) [17]. Furthermore, they also regulate genes that mold the conditions for cellular proliferation. miR-17-92 blocks the production of transforming growth factor (TGF)-β-dependent antiangiogenic factors through suppressing the TGF-β receptor II (TGFBRII), Smad2, and Smad4 [18] whereas miR-18a and miR-19 promotes angiogenesis by directly targeting thrombospondin 1 (TSP-1) and connective tissue growth factor (CTGF), respectively [19].

Iron is essential for various cellular processes, including cell proliferation and growth. It plays a crucial role in DNA synthesis as it is mandatory for the iron-dependent enzyme ribonucleotide reductase that catalyzes the formation of deoxyribonucleotides from ribonucleotides [20], as well as for the regulation of molecules involved in cell cycle control such as p53, GADD45, and CDKN1A [21–24]. Cellular iron availability is regulated by a growing network of genes that maintain cellular iron acquisition, storage, utilization, and export [25]. Among these, ferroportin (FPN) is the only known mammalian iron exporter. It is predominantly expressed in cell types that export iron to the blood stream, including duodenal enterocytes that take up iron from the diet, iron-recycling macrophages, and hepatocytes that store iron. In addition, most cell types express low levels of FPN, including cancer cells [26].

FPN expression is regulated at the transcriptional level by hypoxia-inducible factor-2alpha (HIF2α) in response to hypoxia and iron deficiency [27] as well as by BACH1 and Nrf2 in response to excess heme or oxidative stress [28, 29]. At the translational level, its expression is controlled by iron regulatory proteins (IRPs) that bind to an iron responsive element (IRE) located in its 5′UTR [30]. At the post-translational level, binding of the hepatic iron hormone hepcidin (HAMP) triggers its internalization, ubiquitination, and subsequent lysosomal degradation [31, 32]. In recent years, several examples emerged that genes involved in maintaining iron homeostasis are controlled by miRNAs [33]. For example, the liver specific miR-122 indirectly regulates expression of HAMP by directly targeting two of its upstream activators: the human hemochromatosis protein (HFE) and hemojuvelin (HJV) [34]. In addition, transferrin receptor 1 (TFR1) is negatively regulated by miR-210 and miR-320 causing decreased transferrin uptake and inhibition of proliferation of lung adenocarcinoma A549 cells [35, 36]. miR-let-7d represses expression of an isoform of divalent metal transporter 1 (DMT1) causing accumulation of iron in endosomes of K562 erythroleukemia cells [37]. Finally, miR-485-3p regulates cellular iron homeostasis by directly targeting FPN [38].

In this study, we show that the FPN-3′UTR contains highly conserved and functional target sites for miR-20a, a member of the miR-17 seed family. Our analyses of high-throughput RNA sequencing datasets from The Cancer Genome Atlas (TCGA) revealed that expression of miR-20a is significantly increased in patients with lung adenocarcinoma (LUAD) and lung squamous cell carcinoma (LUSC) and correlates with decreased expression of FPN. Overexpression or knockdown of miR-20a in the non-small cell lung cancer cell lines (NSCLC) H1299 and H1650 significantly affects FPN expression post-transcriptionally. Importantly, low FPN expression stimulates proliferation and colony formation of NSCLC cells, possibly by increasing iron availability for cancer cell proliferation.

## Materials and methods

### Cell culture and reagents

Huh7 and NSCLC cell lines were cultured in DMEM medium (Invitrogen), supplemented with 10 % FBS (Invitrogen). Cell cultures were maintained at 37 °C under 5 % CO_2_. Hepcidin (HAMP) was applied at the concentration of 1 μg/mL (peptide institute).

### RNA isolation, reverse transcription, and quantitative real-time PCR

Total RNA including miRNA was isolated using the miRNeasy Micro Kit (Qiagen) according to the manufacturer’s instructions. mRNAs were reverse transcribed to complementary DNA (cDNA) using the RevertAid RT Reverse Transcription Kit (Thermo Scientific), and miRNAs were reverse transcribed using the miScript II RT kit (Qiagen). Quantification of mature miRNA was performed by using the miScript SYBR-Green PCR kit (Qiagen). SYBR green real-time PCR was performed to quantify mRNA expression levels using the ABI StepOne Plus Real-Time PCR system (Applied Biosystems). mRNA and miRNA expression was calculated relative to ACTB and RNU6, respectively. Data were analyzed using the ΔΔCt method [39]. The primers used are listed in the Table S1.

### Plasmid cloning and site-directed mutagenesis

To generate the pcDNA FPN-enhanced green fluorescent protein (EGFP) construct, the complete human FPN coding sequence was amplified from cDNA of HEK293 cells and inserted into the NheI-XheI restriction sites of the pEGFP-N1 vector (Clontech). To generate the pcDNA FPN-flag construct, the EGFP sequence at the EcoRI-NotI site of the pEGFP-N1 vector was replaced by double-stranded DNA oligo nucleotides containing the flag sequence. Primer sequences are listed in Table S2. The complete 3′UTR segments of the human HIF1A and FPN were amplified by PCR from genomic DNA of Huh7 cells and inserted into the pmirGLO Dual-Luciferase miRNA Target Expression Vector (Promega). In addition, a double-stranded DNA oligo nucleotide encompassing the complete 3′UTR of RPL19 was directly inserted into the vector (pMIR-RPL19). To generate a positive (pMIR-20a^+^) and a negative (pMIR-20a^−^) control vector, a double-stranded DNA oligo nucleotide with the identical sequence of miR-20a was inserted into the pmirGLO vector either in the sense (+) or antisense (−) orientation. Double-stranded DNA oligo nucleotides were annealed in oligo annealing buffer (Promega). Primer sequences are listed in Table S3. Site-directed mutagenesis of the predicted miR-20a seed sequence within the 3′UTR of the genes of interest was performed using the QuikChange II XL Site-Directed Mutagenesis Kit (Agilent Technologies) following the manufacturer’s protocol. Primers are listed in Table S4. All the constructs were confirmed by plasmid DNA sequencing.

### Dual-luciferase reporter assay

Huh7 cells (5 × 10^3^ cells/well) were plated on a sterile 96-well assay plate (Corning), and after 24 h, cells were transfected with 50 nM of miR-20a mimic or a negative control (NC) (Ambion) using RNAiMAX reagent (Invitrogen). After 24 h, 10 ng of luciferase constructs were transfected using Lipofectamine 2000 reagent (Invitrogen). Twenty-four hours later, cells were lysed using 1× passive lysis buffer (Promega), and cellular extracts were analyzed for luciferase activity using the Dual Luciferase Reporter assay system (Promega) and the Centro LB 960 luminometer (Berthold Technologies).

### Cell proliferation and colony-forming assay

To determine proliferation, 30 × 10^3^ cells per well were seeded in a 24-well culture plate in complete growth media. Following trypsinization, cell numbers were determined at the interval of 24 h for 6 days in a hemocytometer. For the colony-forming assay, H1299 cells (1 × 10^3^) were seeded in a 6-well culture plate in complete growth medium. After incubation for 15 days, the colonies were fixed with methanol for 5 min and stained with crystal violet for 15 min, colonies were counted using the Cell Counter v.2.1, a colony counting software.

### Western blot analysis and flow cytometry

Cells were harvested and lysed on ice for 30 min in RIPA buffer supplemented with protease inhibitors (Roche). Antibodies directed against ACTB (Sigma) and FLAG (Sigma) were used. Protein concentration was determined using the DC protein assay (BioRad). Western blot images were acquired using the Fusion-Fx system (Vilber Lourmat). Flow cytometry analysis was done using the BD LSRFortessa cell analyzer (BD Biosciences).

### miRNA expression atlas, target prediction, and TCGA RNA-seq datasets

miRNA expression atlas was generated using MiRZ (https://www.mirz.unibas.ch) [40]. Potential miRNA targets were predicted and analyzed using four publically available algorithms: miRanda (http://www.microrna.org), TargetScan (http://www.targetscan.org), PicTar (http://pictar.mdc-berlin.de), and RNA22 (https://cm.jefferson.edu/rna22/). TCGA RNA-seq datasets from cancer subtypes including LUAD, LUSC, breast cancer (BRCA), and liver hepatocellular carcinoma (LIHC) were downloaded from the publically available UCSC Cancer Genomics Browser (https://genome-cancer.ucsc.edu/) [41].

### Transfection of siRNAs, miRNA mimics/inhibitors, and plasmids

FPN short interfering RNAs (siRNAs) (Ambion), miR-20a mimics, or inhibitors (Ambion) were transfected using Lipofectamine RNAiMAX Reagent (Invitrogen), while plasmid vectors (pmirGLO, pcDNA) were transfected using the Lipofectamine 2000 reagent (Invitrogen).

### Statistical analysis

Data are represented as means ± SEM of at least three independent experiments. The results were analyzed using Prism v.6 (GraphPad). Two-tailed Student’s *t* test was applied for testing the significance for quantitative real-time PCR (qRT-PCR) data and colony formation assays and the two-way ANOVA test for cell proliferation. Pearson’s correlation coefficient was applied to analyze the correlation between expression levels of miRNAs and FPN. Values were considered significantly different when *P* < 0.05.

## Results

### Identification of conserved target sites for members of the miR-17 seed family within the FPN-3′UTR

**Fig. 1:**
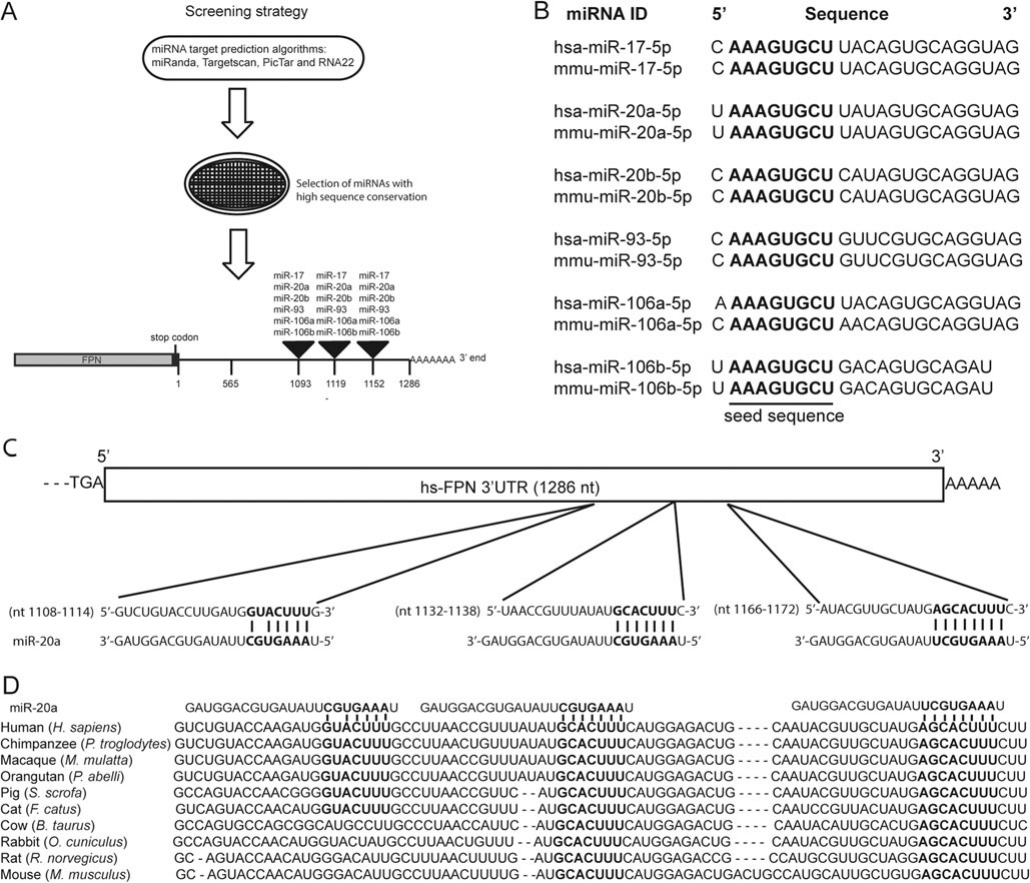
Identification of highly conserved target sites for members of the miR-17 seed family within the FPN-3′UTR. **a** Schematic illustration of the selection process of miRNAs that target the FPN-3′UTR. **b** Members of the human and mouse miR-17 seed family possess highly conserved seed sequences (*bold faced*). **c** miR-20a binding sites reside at nt 1108–1114, nt 1132–1138, and nt 1166–1172 of the FPN-3′UTR (*bold faced*). **d** Sequence alignment between the miR-20a seed sequence and its target sites (*bold faced*) within the FPN-3′UTR of ten mammalian species

To investigate whether the FPN-3′UTR contains miRNA binding sites, we screened the entire 3′UTR sequence of human FPN (1286 nt) using a combination of miRNA target prediction algorithms: miRanda [42], Targetscan [43], PicTar [44], and RNA22 [45]. We next selected only those miRNAs that showed conserved homologues in human and mouse and that were predicted by all algorithms applied. Among these, we identified three putative miR-17 family target sequences (Fig. 1a). The miR-17 family consists of six miRNA members (miR-17, miR-20a/b, miR-93, and miR-106a/b) all sharing conserved seed sequences between human and murine homologues (Fig. 1b). As a representative of the miR-17 family, we chose miR-20a for subsequent investigations. miR-20a target sequences within the human FPN-3′UTR are located at nt 1108–1114, nt 1132–1138, and nt 1166–1172 (Fig. 1c). Interestingly, two of the putative miR-20 binding sites within the FPN-3′UTR sequence are highly conserved among ten species, while the miR-20a target sequence at nt 1108–1114 is conserved among six species (Fig. 1d). Together, these bioinformatic analyses suggest that miRNA members of the miR-17 family might target highly conserved binding sites within the FPN-3′UTR.

### miR-20a downregulates FPN expression by directly targeting the FPN-3′UTR

**Fig. 2:**
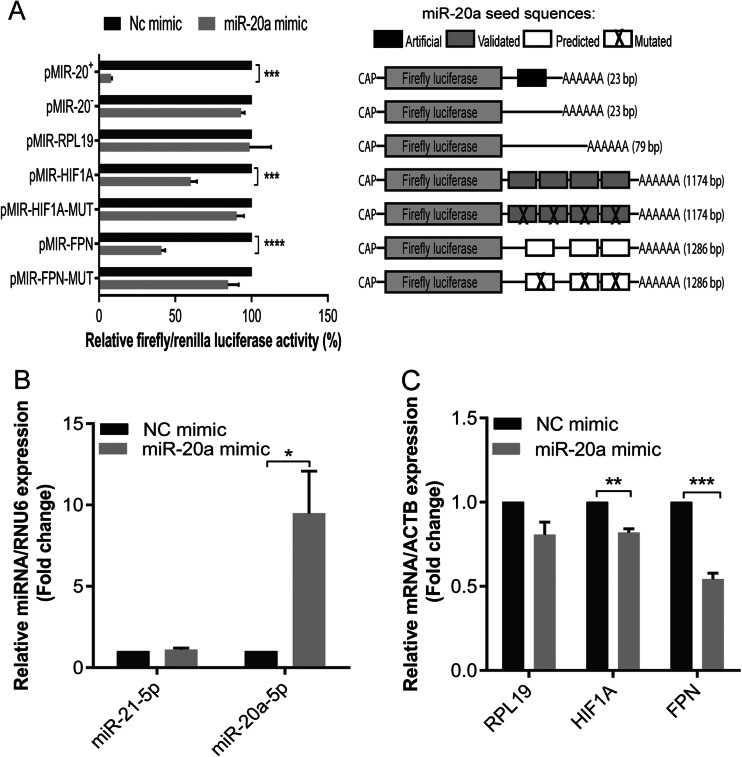
Validation of FPN as a target gene of miR-20a. Huh7 cells were transfected with 50 nM of miR-20a mimic or a negative control (NC). Twenty-four later, **a** the luciferase reporter constructs pMIR-20^+^ (miR-20a complementary sequence), pMIR-20^−^ (identical sequence of miR-20a), pMIR-RPL19, pMIR-HIF1A, pMIR-HIF1A-MUT, pMIR-FPN, or pMIR-FPN-MUT were transfected (*MUT* mutated miR-20a or HIF1A target sites, see Fig S1). Luciferase activity was measured 48 h later. **b** To test specific and efficient transfection of miR-20a, we measured miR-21 and miR-20a levels by qPCR. **c** mRNA expression of RPL19 (negative control), HIF1A (positive control), and FPN were measured by qPCR. Experiments were performed in triplicates and repeated at least three times. Data were normalized to appropriate reference genes: RNU6 (**b**) and ACTB (**c**). Data are represented as mean ± SEM, and the values from negative control (NC) mimic were set to 100 % (**a**) or 1 (**b**, **c**) *P < 0.05, **P < 0.001, ***P < 0.001, ****P < 0.0001, two-tailed Student’s *t* test

To investigate whether miR-20a directly targets FPN by binding to the bioinformatically predicted miR-20a binding sites within its 3′UTR, we generated luciferase reporter constructs bearing the full 3′UTR sequence of human FPN (referred herein as pMIR-FPN) or constructs with mutated miR-20a target sites (Fig. S1B) to assess the specificity of miR-20a-dependent FPN regulation. As a negative control, we inserted the complete 3′UTR of the human RPL19 gene within the same vector (pMIR-RPL19), which does not have predicted miR-20a target sites. As a positive control, we cloned the 3′UTR of the human HIF1A gene (pMIR-HIF1A), which is a validated miR-20a target, or of a mutant derivative (Fig. S1A) [46]. In addition, artificial positive and negative control vectors with perfect sequence complementary to miR-20a or identical to miR-20a (pMIR-20^+^ and pMIR-20^−^, respectively) were studied. miR-20a mimics were transfected together with the luciferase reporter plasmids into Huh7 human hepatocarcinoma cells. Upon miR-20a overexpression, luciferase activity from cells transfected with the positive control plasmids pMIR-20^+^ and pMIR-HIF1A was strongly reduced whereas luciferase activity was unaffected from cells transfected with the negative control plasmids pMIR-20^−^, pMIR-HIF1A-MUT, or pMIR-RPL19 (Fig. 2a), suggesting that miR-20a overexpression is efficient and specific. Importantly, overexpression of the miR-20a mimic significantly reduced luciferase activity of pMIR-FPN but not of pMIR-FPN-MUT, in which predicted miR-20a target sites were mutated (Fig. 2a). To further investigate whether overexpression of miR-20a negatively regulates endogenous FPN mRNA expression, we analyzed FPN mRNA levels from miR-20a mimic-transfected Huh7 cells. We show that mRNA levels of FPN and HIF1A (positive control) were significantly reduced upon miR-20a overexpression (Fig. 2b) whereas mRNA levels of RPL19 (negative control) remained unaffected (Fig. 2c). Taken together, our data show that miR-20a directly targets the FPN-3′UTR and negatively regulate FPN mRNA levels.

### Expression of miR-20a is inversely correlated to FPN in lung cancer

**Fig. 3:**
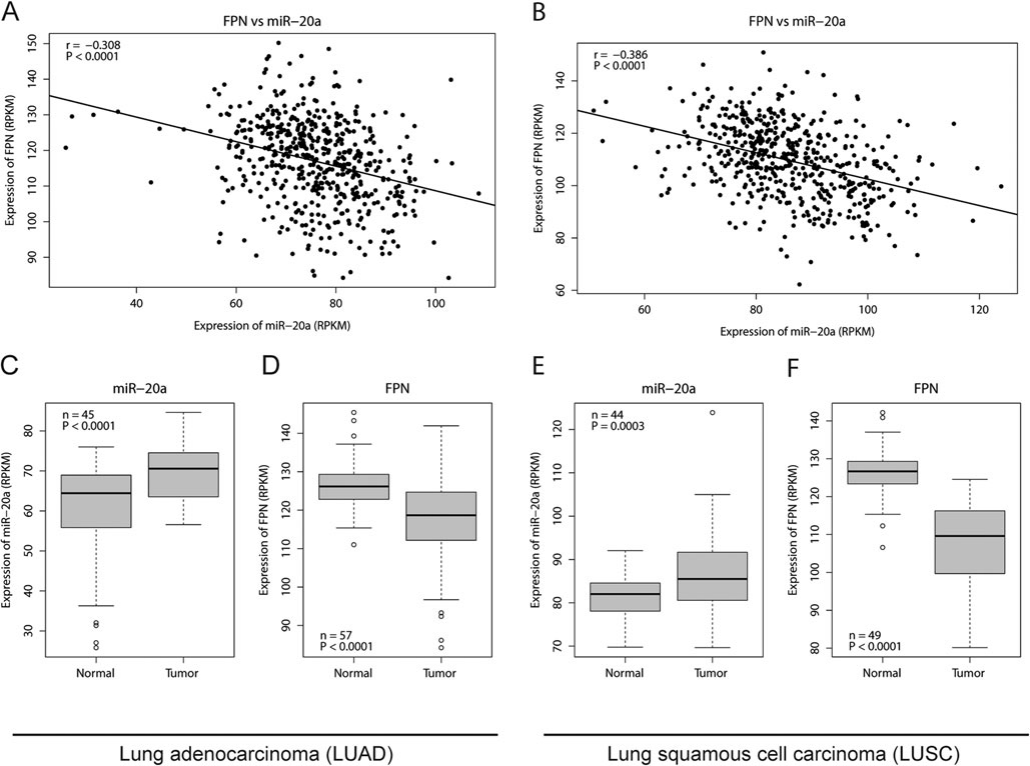
mRNA expression of FPN is inversely correlated to the expression of miR-20a in lung cancer. TCGA RNA-seq datasets of lung adenocarcinoma (LUAD) and lung squamous cell carcinoma (LUSC) patients were downloaded from the UCSC cancer genomic browser (https://genome-cancer.ucsc.edu) [41]. Correlations between miR-20a and FPN expression levels are represented as scatter plots for **a** LUAD (*n* = 524, *r* = −0.308, *P* < 0.0001) and **b** LUSC (*n* = 513, *r* = −0.386, *P* < 0.0001), Pearson’s product moment correlation was used. Box plots illustrate that in patients with LUAD, **c** miR-20a is significantly elevated (*n* = 45, *P* < 0.0001) while **d** FPN mRNA expression is significantly decreased (*n* = 57, *P* < 0.0001) compared to matched normal healthy tissues. Similarly, in patients with LUSC, **e** miR-20a is significantly increased (*n* = 44, *P* = 0.0003) whereas **f** FPN is significantly decreased (*n* = 49, *P* < 0.0001), two-tailed Student’s *t* test, *RPKM* reads per kilobase per million mapped reads

We next explored the clinical relevance of interactions between miR-20a, a member of the oncogenic miR-17-92 cluster that is frequently deregulated in cancer [13], and FPN by correlating RNA expression data from cancer patients. We limited our analysis to high-quality RNA-seq data sets available within TCGA (http://cancergenome.nih.gov/) from patients with LUAD, LUSC, BRCA, and LIHC. RNA-seq datasets was chosen to be able to differentiate the expression of each miRNA member of the highly conserved miR-17 family. We found that mRNA expression of FPN is significantly inversely correlated with that of miR-20a (*P* < 0.0001) in LUAD patients (*n* = 524) (Fig. 3a) and in the LUSC patients (*n* = 513) (Fig. 3b). Our analysis further revealed that in LUAD, the expression of miR-20a was significantly elevated (*P* < 0.0001) in tumor compared to matched healthy tissue from the same patient (Fig. 3c). Conversely, the mRNA expression of tissue FPN was significantly decreased (*P* < 0.0001) (Fig. 3d). Similar results were obtained by comparing tumor samples of LUSC patients, in which miR-20a was significantly overexpressed (*P* = 0.0003) in tumor tissue (Fig. 3e), while FPN mRNA expression was significantly decreased in matching healthy tissue (*P* < 0.0001) (Fig. 3f). Moreover, the expression of other miR-17 family members, except of miR-20b was also significantly increased in tumors of LUAD and LUSC patients (Fig. S2). Correlation analyses of FPN mRNA expression and expression levels of miR-17 seed family members revealed that in LUAD patients, FPN mRNA expression was negatively correlated to all six miRNAs of the miR-17 family (Fig. 3a and S3A). By contrast, in LUSC and in LIHC, FPN mRNA expression was significantly negatively correlated to miR-17, miR-20a, miR-106b, and miR-93 expression levels but not to miR-106a and miR-20b (Fig. 3b, S3B and S4A). Likewise, in BRCA, we found that FPN expression was negatively correlated to miR-17 miRNA members except miR-20b (Fig. S4B). Collectively, these data suggest that overexpression of miR-20a might be involved in regulating FPN mRNA levels in several cancer subtypes, including lung cancer.

### FPN is post-transcriptionally regulated by miR-20a in NSCLC

**Fig. 4:**
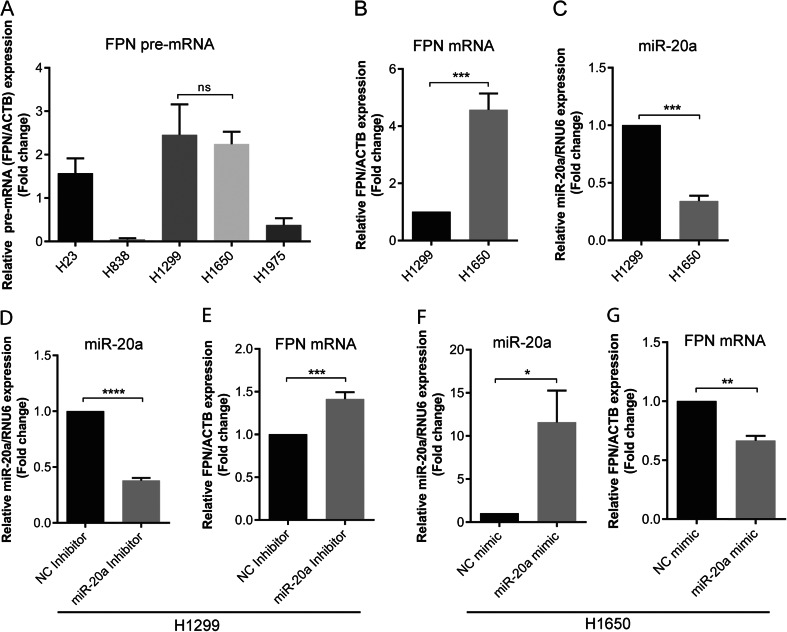
FPN is post-transcriptionally regulated by miR-20a in non-small cell lung cancer (NSCLC) cells. **a** A total of five NSCLC were analyzed for FPN pre-mRNA levels by qPCR. Insignificant differences of FPN pre-mRNA expression were only observed between H1299 and H1650 (*P* = 0.7981). **b** FPN mRNA expression and **c** miR-20a expression in the H1299 and H1650 cell lines. H1299 cell were transfected with 50 nM of miR-20a inhibitors or a negative control (NC). Twenty-four hours later, the expression of miR-20a (**d**, **f**) and FPN (**e**, **g**) mRNA were analyzed by qPCR. Experiments were performed in triplicates and repeated at least three times. Data were normalized to appropriate reference genes: ACTB (**a**, **b**, **e**, **g**) or RNU6 (**c**, **d**, **f**). Data are represented as mean ± SEM, and the values from negative controls (NC) mimic or inhibitor were set to 1. *ns* no statistical significance, *P < 0.05, **P < 0.01, ***P < 0.001, ****P < 0.0001, two-tailed Student’s *t* test

To further explore the relationship of miR-20a and FPN expression in lung cancer, a total of five NSCLC cell lines (H23, H838, H1299, H1650, and H1975) were analyzed for the expression of premature mRNA (pre-mRNA) of FPN to identify cell lines that show comparable levels of FPN transcription. We show that pre-mRNA levels of FPN in the five NSCLC cell lines differed significantly, whereby FPN pre-mRNA expression (*P* = 0.7981) was similar between H1299 and H1650 cells (Fig. 4a). Analysis of FPN mRNA and miR-20a expression in the H1299 and H160 cell lines showed that expression of FPN mRNA in H1650 was significantly higher compared to H1299 (Fig. 4b) whereas the inverse result was obtained for miR-20a (Fig. 4c). Next, we examined whether manipulation of miR-20a levels could affect the expression of FPN mRNA in NSCLC cell lines. We transfected miR-20a inhibitors into H1299 or miR-20a mimics into H1650 in order to reduce or increase miR-20a levels, respectively, and then, we analyzed the levels of miR-20a and FPN mRNA by qPCR. We observed that miR-20a levels were significantly reduced in miR-20a inhibitor-transfected H1299 cells (Fig. 4d) confirming successful knockdown of miR-20a. At the same time expression of FPN mRNA was significantly increased (Fig. 4e). Likewise, levels of miR-20a were significantly increased in the miR-20a mimic-transfected H1650 cells (Fig. 4f) implying successful overexpression of miR-20a, whereby FPN mRNA expression was reduced (Fig. 4g). These results suggest that miR-20a regulates FPN expression post-transcriptionally in NSCLC.

### miR-20a and FPN expression control NSCLC proliferation and colony formation

**Fig. 5:**
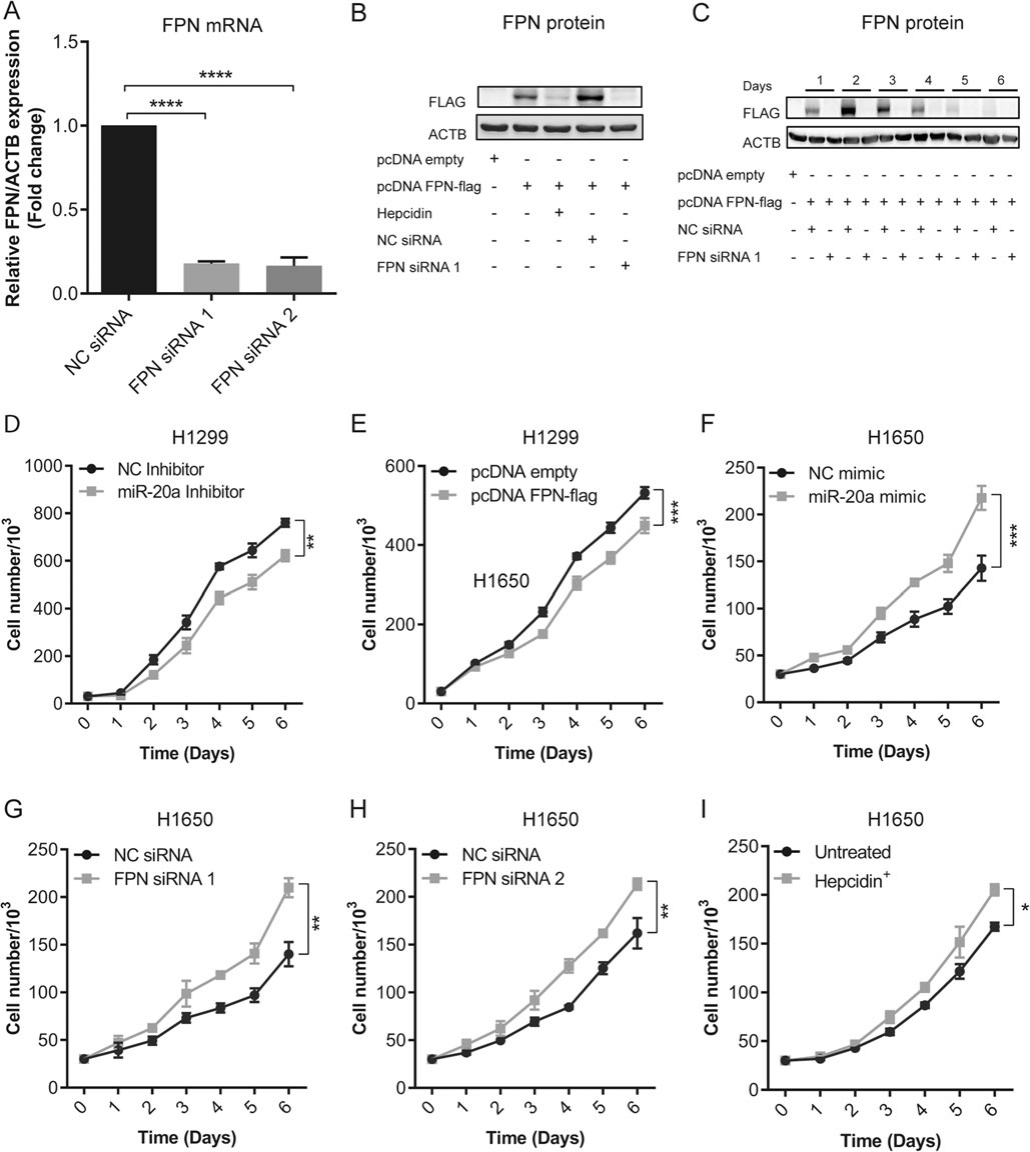
Alteration of miR-20a or FPN expression affects NSCLC proliferation. H1299 cells were transiently transfected with either miR-20a inhibitor, a negative control (NC), the expression plasmid pcDNA FPN-flag, or a control vector (pcDNA empty). H1650 cells were transiently transfected with either miR-20a mimic, a negative control (NC), specific FPN siRNAs (FPN siRNA 1 and FPN siRNA 2) or NC siRNA. **a** FPN mRNA levels analyzed by qPCR in H1650 cells. qPCR data were normalized to ACTB. ****P < 0.0001, two-tailed Student’s *t* test. **b**, **c** Western blot analysis of FPN protein levels following RNAi or overexpression of FPN in H1299 cells. **c** Time course experiment showing the effects of FPN overexpression or knockdown on cell proliferation for up to 5 days. **d**–**i** Proliferation assays performed in H1299 cells (**d**, **e**) or H1650 cells (**f**–**i**) in response to the indicated treatments. All experiments were performed in triplicates and repeated at least three times. Data represented as mean ± SEM. Two-way ANOVA was applied to calculate statistical significance of proliferation curves. *P < 0.05, **P < 0.01, ***P < 0.001

We next assessed the potential pathological relevance of miR-20 and FPN expression in NSCLC. Previous studies showed that overexpression of miR-20a promotes lung cancer cell proliferation [47] and that overexpression of FPN reduces breast tumor growth in mice [48]. Thus, we hypothesized that expression levels of miR-20a and FPN could alter the rate of cell proliferation. To manipulate miR-20a levels, we either transiently transfected miR-20a inhibitors or miR-20a mimics. FPN expression was increased by transfecting the vector pcDNA FPN-flag into H1299 cells, which expresses a flag-tagged FPN (Fig. 5b). Expression of a tagged FPN protein version is required, as sensitive antibodies directed against human FPN are unavailable. The transfection rate of H1299 cells is close to 100 %, as was estimated by transiently transfecting H1299 cells with a vector in which FPN is fused to EGFP (pcDNA FPN-EGFP) (Fig. S5). Importantly, overexpressed FPN is localized to the cell membrane in pcDNA FPN-flag transfected H1299 cells, because treatment with the iron hormone hepcidin efficiently reduces FPN protein levels (Fig. 5b).

The proliferation rate of transiently transfected cells was analyzed by counting cell numbers in a hemocytometer for 6 days. We show that inhibition of miR-20a in H1299 cells represses cell proliferation (*F*(6,28) = 4.157; *P* = 0.0041) (Fig. 5d), while in miR-20a mimic-transfected H1650 cells, proliferation was significantly increased (*F*(6,28) = 7.153; *P* = 0.0001) (Fig. 5f). Furthermore, FPN-flag overexpression in transfected H1299 cells decreased cell proliferation (*F*(6,28) = 5.369; *P* = 0.0009) (Fig. 5e). By contrast, transfection of two different siRNAs that efficiently reduce FPN mRNA and protein levels (Fig. 5a–c) increased cell proliferation of H1650 cells: FPN siRNA1 (*F*(6,28) = 5.047; *P* = 0.0013) (Fig. 5g) and FPN siRNA2 (*F*(6,28) = 4.335; *P* = 0.0033) (Fig. 5h). Similar results were obtained when FPN protein levels were reduced by treatment with HAMP (1 μg/mL) in the growth medium (*F*(6,28) = 3.393; *P* = 0.0121) (Fig. 5i).

Reduced FPN expression decreases the capacity for iron export and causes cellular iron retention. As a marker for cellular iron content, we analyzed mRNA levels of transferrin receptor (TFR1). Its expression is regulated by the IRP/IRE system [49] in that mRNA levels are increased by cellular iron deficiency and decreased when cellular iron levels are elevated. TFR1 mRNA expression in H1650 cells either transfected with miR-20a mimic or siRNA directed against FPN is significantly reduced in a time-dependent manner (Fig. S7), suggesting that the observed reduction in FPN mRNA levels causes reduced iron export and iron accumulation in cells.

**Fig. 6:**
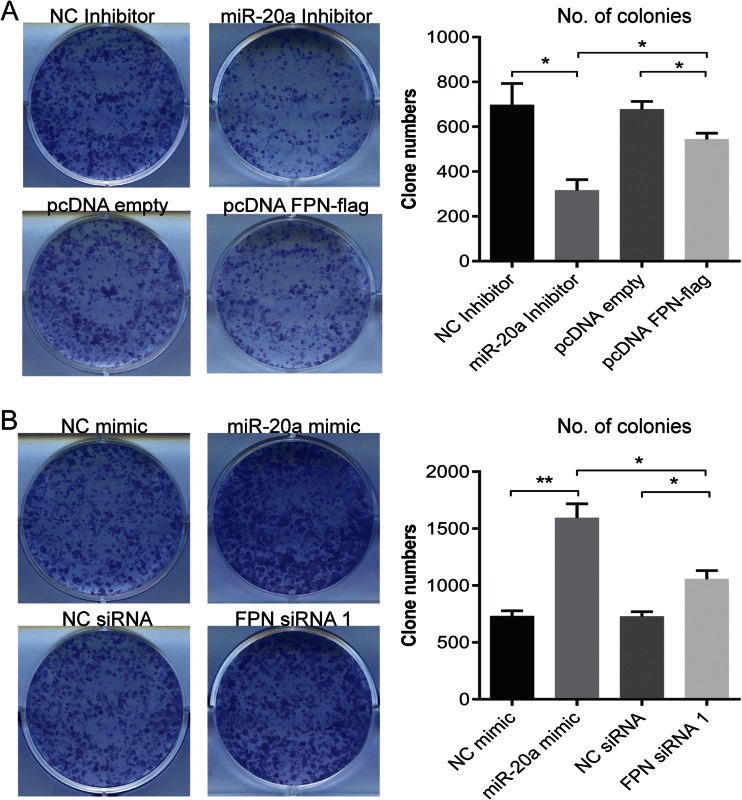
Colony formation of H1299 cells is affected by expression levels of miR-20a and FPN. H1299 cells were transiently transfected with either a miR-20a mimic, a miR-20 inhibitor, pcDNA FPN-flag, or FPN siRNA. Twenty-four hours later, the cells were seeded at a clonal density of 1000 cells/well in a 6-well plate. After 15 days of incubation, the colonies were counted using the Cell Counter v.2.1. Quantitative data are shown in **a** and **b**. Treatments are indicated. *Left*, representative pictures of colonies formed; *right*, quantitation of colonies. Experiments were performed in triplicates and repeated at least three times. Data were presented as mean ± SEM. *P < 0.05, **P < 0.01, two-tailed Student’s *t* test

By applying similar experimental approaches, we next assessed whether miR-20a and FPN expression levels can affect NSCLC colony formation. H1299 cells were transiently transfected with miR-20a mimics/inhibitors or pcDNA FPN-flag/FPN siRNA, respectively. Twenty-four hours later, the transfected cells were seeded in 6-well plates at a defined clonal density (1000 cells/well), and 15 days later, the number of colonies were counted. Interestingly, both the knockdown of miR-20a and overexpression of FPN-flag significantly decreased the capacity to form colonies, whereby the knockdown of miR-20a showed a stronger effect compared to FPN-flag overexpression (*P* = 0.0152) (Fig. 6a). Vice versa, overexpression of miR-20a and RNAi of FPN significantly increased the colony-forming capacity, whereby overexpression of miR-20a showed stronger effects compared to RNAi of FPN (*P* = 0.0205) (Fig. 6b). Taken together, our data show that miR-20a controlled FPN expression contributes to increase cellular proliferation as well as the capacity of NSCLC to form colonies.

## Discussion

This study identifies FPN as a target of miR-20a. miR-20a-dependent FPN regulation enhances the rate of cancer cell proliferation and the capacity to form colonies. The FPN 3′UTR contains three evolutionarily conserved, functional target sites for the highly homologous miR-17 seed family miRNA members (Fig. 1). Two miR-17-5p target sites at nt 1132–1138 and nt 1116–1172 within the FPN-3′UTR were predicted by a recent study, but the functionality was not proven [50]. The same study predicted a target site for miR-17-3p, the passenger strand of miR-17 at nt 61–67, suggesting that FPN may also be targeted by passenger strands of miR-17 seed family members. Another study showed that cellular iron deprivation induces miR-485-3p, which regulates FPN by directly targeting sites within the FPN-3′UTR at nt 448–454 and nt 618–625 [38]. These findings suggest that miRNAs may play important roles in regulating FPN expression in physiological and pathophysiological condition. This adds an additional level of complexity to the control of FPN expression, which was previously shown to be regulated at the transcriptional, translational, and post-translational level [27–29, 31, 32, 51].

Here, we demonstrate for the first time that elevated miR-20a levels suppress expression of endogenous FPN mRNA in cell lines (e.g., Huh7 and H1650) (Fig. 2c and 4g), while reduced miR-20a levels increase expression of endogenous FPN mRNA (Fig. 4e). Unfortunately, sensitive antibodies that detect human FPN are unavailable, hampering analysis of endogenous FPN protein expression. We therefore validated the bioinformatically predicted miRNA target sites by transfecting cells with luciferase reporter constructs that contain the entire FPN-3′UTR. Our experiments show that luciferase activity is altered in a miR-20a-dependent manner that requires the predicted target sequences within the FPN-3′UTR (Fig. 2a).

Our analysis further uncovered a significant negative correlation between mRNA expression levels of FPN and members of the miR-17 seed family in several cancer subtypes, including lung, breast, and liver cancer (Fig. 3, S3, S4). While correlations were strong for most miR-17 seed family members, including miR-20a, it was not apparent for miR-106a and miR-20b, which are expressed from the miR-106a-363 cluster at very low levels [52]. These findings suggest that miR-20a and miRNAs with identical seed sequences reduce levels of the iron exporter FPN in various human cancer subtypes. We further observe that FPN levels are decreased in two lung cancer subtypes (LUAD and LUSC; Fig. 3d, f, respectively) compared to healthy lung tissue. This is reminiscent of observations in breast cancer, colorectal cancer, and multiple myeloma. In addition, lower expression of FPN is linked to poor survival in breast cancer and in multiple myeloma [48, 53–55]. Our results extend upon these observations by demonstrating that manipulation of FPN levels directly or mediated by alteration of miR-20a or treatment with hepcidin affects cancer cell proliferation (Fig. 5) and colony formation (Fig. 6). It is of note that in our cellular assays, changes in miR-20a levels triggered by transfection with either miR-20a inhibitor or mimic are more pronounced than those observed between normal and tumor tissues in TCGA RNA-sequencing datasets of LUAD and LUSC (Fig. 3c–f). Whether the small changes in FPN mRNA levels in tumor tissues are sufficient to alter FPN protein expression and iron metabolism is not clear.

In addition to FPN, miR-20a targets additional genes, which are involved in cell cycle control and growth [13]. This is evidenced by the fact that miR-20a manipulation affects colony formation more strongly compared to direct alterations of FPN levels (Fig. 6). This suggests that differences of miR-20a expression will affect FPN plus additional target genes involved in cell cycle and growth causing cumulative effects on cell proliferation and colony formation (Figs. 5 and 6).

Furthermore, the oncogenic miR-20a may control iron metabolism indirectly, e.g., by modulating the expression levels of the transcription factor HIF1A (Fig. 2a, c) [46, 56]. As the hypoxic response and regulation of iron metabolism are tightly interconnected [57], HIF1A may be involved in regulating genes that maintain iron homeostasis, e.g., transferrin, TFR1, ceruloplasmin, and heme oxygenase 1 in cancer [58–64]. miR-17 family members also directly target the amyloid precursor protein (APP) [65, 66] that was hypothesized to exert ferroxidase activity and to facilitate iron export by stabilizing FPN [67, 68].

Two different miRNAs, miR-20a and miR-485, control FPN expression (data shown here and [38]). Therefore, we additionally analyzed miR-485 and FPN expression in RNA sequencing data from LUAD and LUSC patients. Similar to our findings with miR-20a, miR-485 expression negatively correlates to FPN mRNA expression (Fig. S6A and B). Despite that miR-485 expression was only significantly increased in tumors of LUSC but not in LUAD (Fig. S6C, E). These findings are consistent with previous reports that show roles for forced miR-485-3p expression in promoting tumorigenesis and metastasis in mice [69], indirectly affecting sensitivity to etoposide and fludarabine by fine tuning DNA Topoisomerase II expression via nuclear factor-YB [70, 71] and by regulating methionine adenosyltransferase 1A expression in hepatocellular carcinoma [69]. It is of note that miR-20a is significantly higher expressed compared to miR-485 in lung cancer (Fig. S6D, F) as well as in most other tissues or cell types analyzed (Fig. S8). This may suggest that miR-485 and miR-20a cooperate in regulating FPN levels in lung cancer, but that the regulatory effect of miR-20a on FPN may dominate over the one of miR-485 due to its low expression level.

How is the alteration of FPN levels linked to cell proliferation and tumor growth? Previous findings showed that changes in FPN expression affect the labile iron pool (LIP) [31, 48, 54] and thus will influence intracellular availability of this important nutrient. In addition, the treatment of cells with iron chelators that limit iron supplies arrest the cell cycle between the G1/S phase or in some instances between the G2/M phase [72–74], suggesting that cell cycle check points exist that monitor iron levels. Iron chelation was therefore tested as an anticancer therapy [26, 75].

Taken together, these findings suggest that manipulation of FPN expression may cause changes in intracellular iron availability in NSCLC cells that influence the rate of cell proliferation. Increased expression of miR-20a, which is observed in diverse tumor entities will decrease FPN levels and retain iron in tumor cells, in addition to regulating numerous other genes involved in cell proliferation and growth [13, 76, 77]. In addition, in cancer cells, other genes involved in maintaining cellular iron homeostasis are differentially expressed. These include genes involved in iron acquisition (e.g., TFR1) [78] or iron storage [79, 80], which are directly or indirectly regulated by oncogenes like MYC or HRAS [81, 82].

In conclusion, our results strongly suggest that the downregulation of FPN by the oncogenic miR-20a, which is overexpressed in various cancer entities, may cause an increase in the cellular labile iron pool thus providing surplus iron for metabolic processes like DNA synthesis or the proliferation or growth of cancer cells. Overall, post-transcriptional regulation of FPN by miR-20a may act as a contributing factor to cancer prognosis making FPN a potential target for anticancer therapy.

## Electronic supplementary material

ESM 1 (PDF 920 kb)
