# Supplementary material for: miR-20a regulates expression of the iron exporter ferroportin in lung cancer
Source: J Mol Med (Berl). 2015 Nov 12;94:347–59. doi: 10.1007/s00109-015-1362-3 (PMC4803811; doi:10.1007/s00109-015-1362-3)
Supplement: Supplementary file 1 — (PDF 920 kb) [file 109_2015_1362_MOESM1_ESM.pdf]

Electronic Supplementary Material

miR-20a regulates expression of the iron exporter ferroportin in lung cancer

Kamesh R. Babu and Martina U. Muckenthaler

Journal of Molecular Medicine 2015

SUPPLEMENTARY MATERIAL:

Name	5' Sequence 3'
hs-ACTB_F	TTCCGCTGCCCTGAGGCACTCT
hs-ACTB_R	TCTGCTGGAAGGTGGACAGCGA
hs-pre-ACTB_F	CTATTCTCGCAGCTCACCATG
hs-pre-ACTB_R	CAGCTCCCCTACCTGGTG
hs-FPN_F	TGCTGTTTGCAGGCGTCATT
hs-FPN_R	TTGCAGCAACTGTGTCACAGTT
hs-pre-FPN_F	TACCCATTGGGAAGGGGAAT
hs-pre-FPN_R	CCAGGGGTTTTGGCTCAGTA
hs-HIF1A_F	CCCCAGATTCAGGATCAGACA
hs-HIF1A_R	GGGACTATTAGGCTCAGGTGAACTT
hs-RPL19_F	TCGCCTCTAGTGTCCTCCG
hs-RPL19_R	GCGGGCCAAGGTGTTTTTC
hs-RNU6	GTGCTCGCTTCGGCAGCACATATAC
hs-miR-20a-5p	TAAAGTGCTTATAGTGCAGGTAGG
hs-miR-21-5p	TAGCTTATCAGACTGATGTTGAGG

Table S1: Primers used for qPCR analysis of mRNA and miRNA transcripts

Name	5’ Sequence 3’
h-FPN-F	AAAGCTAGCATGACCAGGGCGGGAG
h-FPN-R	AAACTCGAGAACAACAGATGTATTTGCTTGATTT
Flag_F	[Phos]AATTCTAGACTACAAGGACGACGATGACAAGTGAGC
Flag_R	[Phos]GGCCGCTCACTTGTCATCGTCGTCCTTGTAGTCTAG

Table S2: Primers used to generate FPN expression constructs. Sequences with 5’phosphate represented as [Phos] were annealed using oligo annealing buffer followed by direct ligating into the vector.

Name	5' Sequence 3'
hs-FPN_F	GAGAGCTCGGAAGGAAAATCAAGCAAATACA
hs-FPN_R	GAGCTAGCTTGGGGAACAGAATTCAGGA
hs-HIF1A_F	GAGAGCTCACTCAGAGCTTTGGATCAAGTT
hs-HIF1A_R	GAGCTAGCGCCTGGTCCACAGAAGATGT
hs-RPL19_F	[Phos]CAACCTCCCACTTTGTCTGTACATACTGGCCTCTGTGATTACATAGATCAGCCATTAAAATAAAACAAGCCTTAATCTGCG
hs-RPL19_R	[Phos]CTAGCGCAGATTAAGGCTTGTTTTATTTTAATGGCTGATCTATGTAATCACAGAGGCCAGTATGTACAGACAAAGTGGGAGGTTGAGCT
hs-miR-20a-5p(+)_F	[Phos]CCTACCTGCACTATAAGCACTTTAG
hs-miR-20a-5p(+)_R	[Phos]CTAGCTAAAGTGCTTATAGTGCAGGTAGGAGCT
hs-miR-20a-5p(-)_F	[Phos]CTAAAGTGCTTATAGTGCAGGTAGG
hs-miR-20a-5p(-)_R	[Phos]CTAGCCTACCTGCACTATAAGCACTTTAGAGCT

Table S3: Primers used for the amplification of 3’-UTR sequences. Sequences with 5’phosphate represented as [Phos] were annealed using oligo annealing buffer followed by direct ligating into the vector.

.

Name	5' Sequence 3'
hs-FPN-MT1_F	TCAGTTTGCAACATGTCTGTACCAAGATGGTAGAATGCCTTAACCGTTTATA
hs-FPN-MT1_R	TATAAACGGTTAAGGCATTCTACCATCTTGGTACAGACATGTTGCAAACTGA
hs-FPN-MT2_F	GATGGTAGAATGCCTTAACCGTTTATATGCAGAATCATGGAGACTGCAATAC
hs-FPN-MT2_R	GTATTGCAGTCTCCATGATTCTGCATATAAACGGTTAAGGCATTCTACCATC
hs-FPN-MT3_F	GGAGACTGCAATACGTTGCTATGAGCAGAATCTTTATCCTTGGAGTTTAAT
hs-FPN-MT3_R	ATTAAACTCCAAGGATAAAGATTCTGCTCATAGCAACGTATTGCAGTCTCC
hs-HIF1A-MT1_F	TGGTATTTAAACCATTGCATTGCAGTAGCATCAGAATAAAAAATGCACCTTTTTATTTATTTATTTTTGG
hs-HIF1A-MT1_R	CCAAAAATAAATAAATAAAAAGGTGCATTTTTTATTCTGATGCTACTGCAATGCAATGGTTTAAATACCA
hs-HIF1A-MT2_F	TACTGCTGGCAAAGCATTATTATTTATGTATTCTGTGAAAAAAAAGGTGTAAAAATTTTTCAACTGCCT
hs-HIF1A-MT2_R	AGGCAGTTGAAAAATTTTTACACCTTTTTTTTCACAGAATACATAAATAATAATGCTTTGCCAGCAGTA
hs-HIF1A-MT3_F	TTTTCTTTTGAGCTGGCATTCTGACTATAGAAACATCAGATGATTTCTCTGAATTG
hs-HIF1A-MT3_R	CAATTCAGAGAAATCATCTGATGTTTCTATAGTCAGAATGCCAGCTCAAAAGAAAA
hs-HIF1A-MT4_F	AGTATGTTAATTTGCTCAAAATACAATGTTTGATTTTATGCAGAATGTCGCTATTAACATCCTTTTTTT
hs-HIF1A-MT4_R	AAAAAAAGGATGTTAATAGCGACATTCTGCATAAAATCAAACATTGTATTTTGAGCAAATTAACATACT

Table S4: Primers used to mutagenize the miR-20a-5p seed sequences

Figure S1

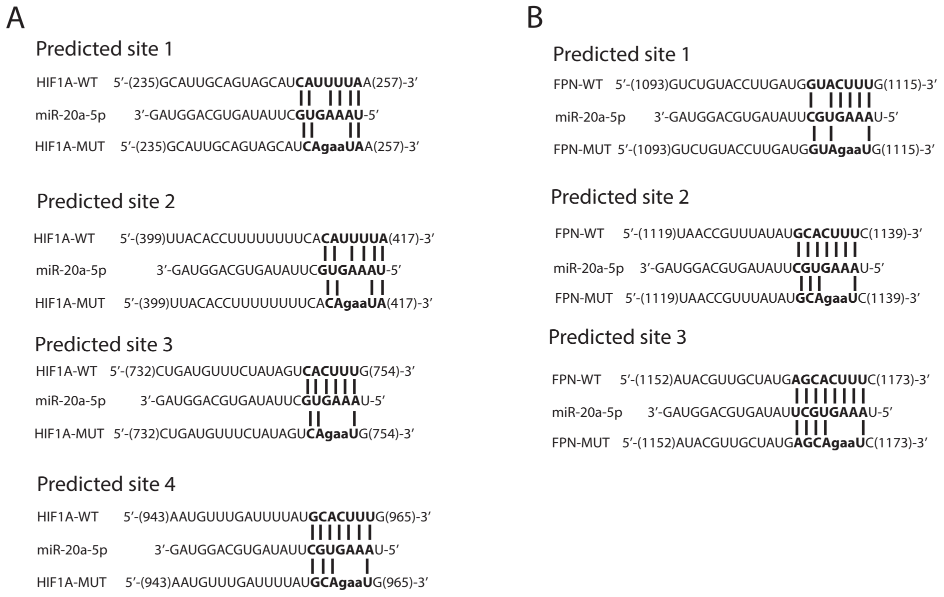

Figure S1. Putative miR-20a binding sites in the 3'UTRs of human (A) HIF1A and (B) FPN. The nucleotides that are mutated in the wild type 3'UTRs are indicated in bold and lower case.

Figure S2

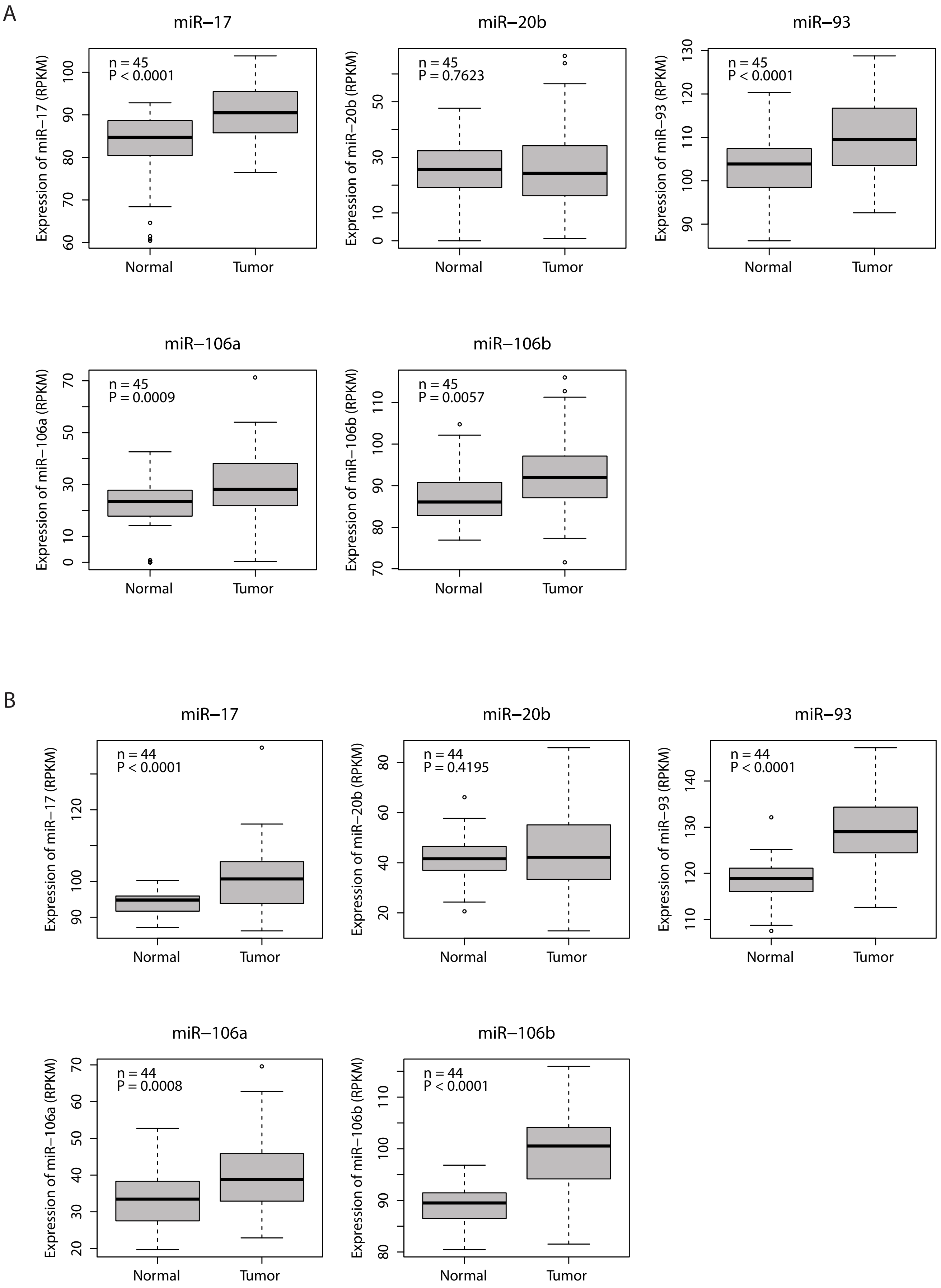

Figure S2. Boxplots indicating expression levels of the indicated member of the miR-17 seed family in the tumor and matching healthy tissue within TCGA datasets of (A) lung adenocarcinoma (LUAD) and (B) lung squamous cell carcinoma (LUSC) patients. 2-tailed Student’s t test was used to calculate statistical significance.

Figure S3

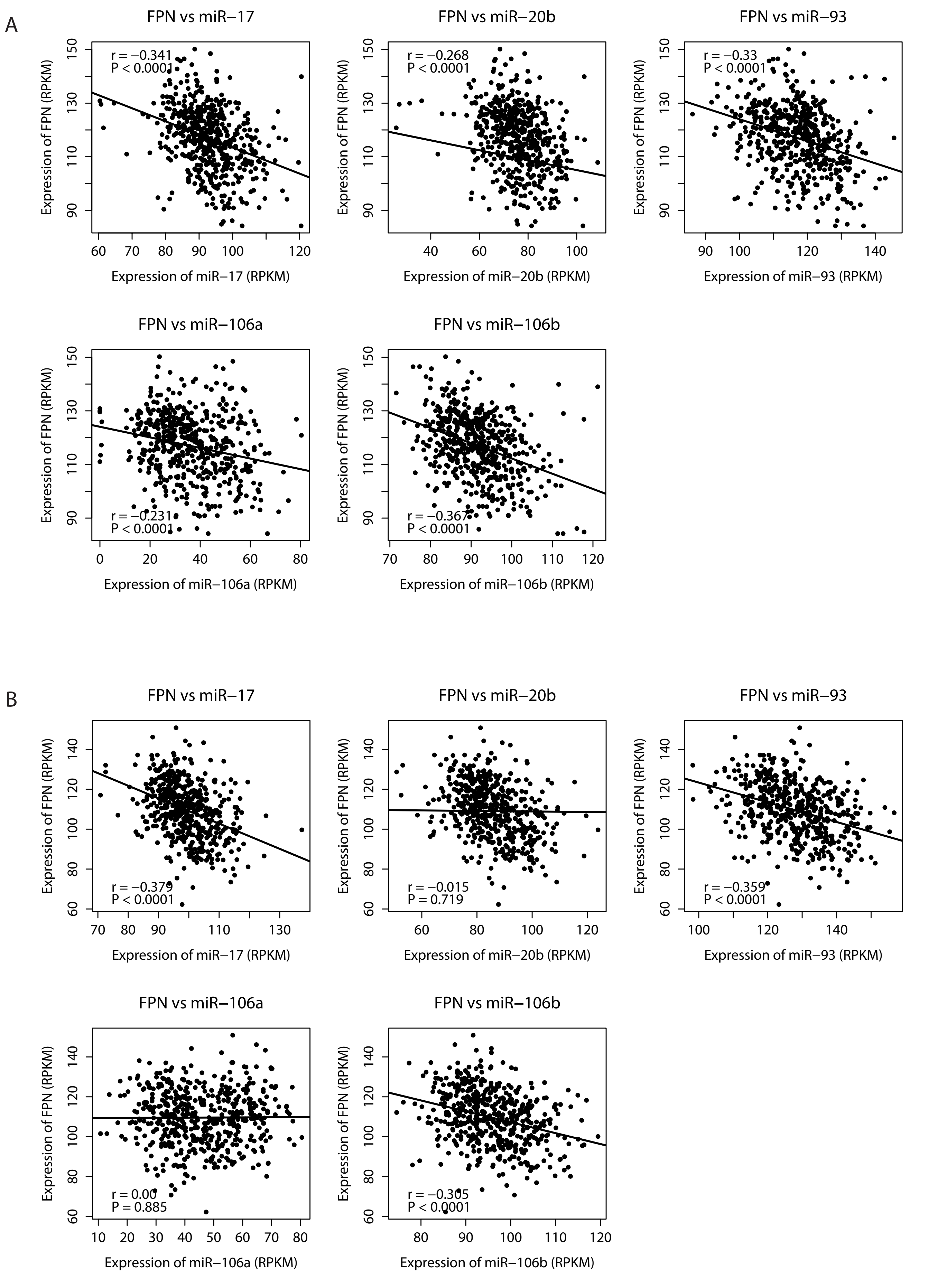

Figure S3. Scatter plots indicating correlations of FPN mRNA levels to the indicated member of the miR-17 seed family within TCGA datasets of (A) lung adenocarcinoma (LUAD) and (B) lung squamous cell carcinoma (LUSC) patients. Pearson's product moment correlation was used to calculate statistical significance.

Figure S4

A

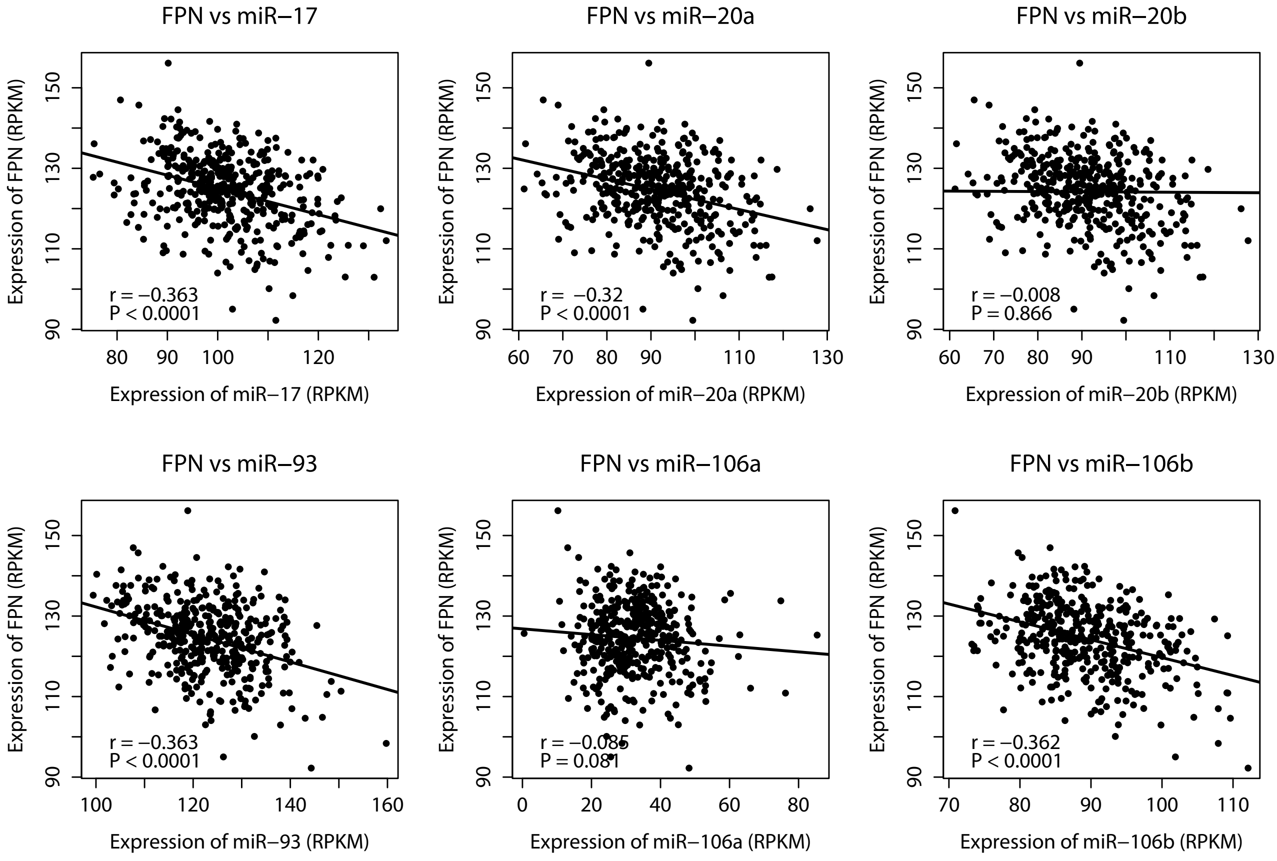

B

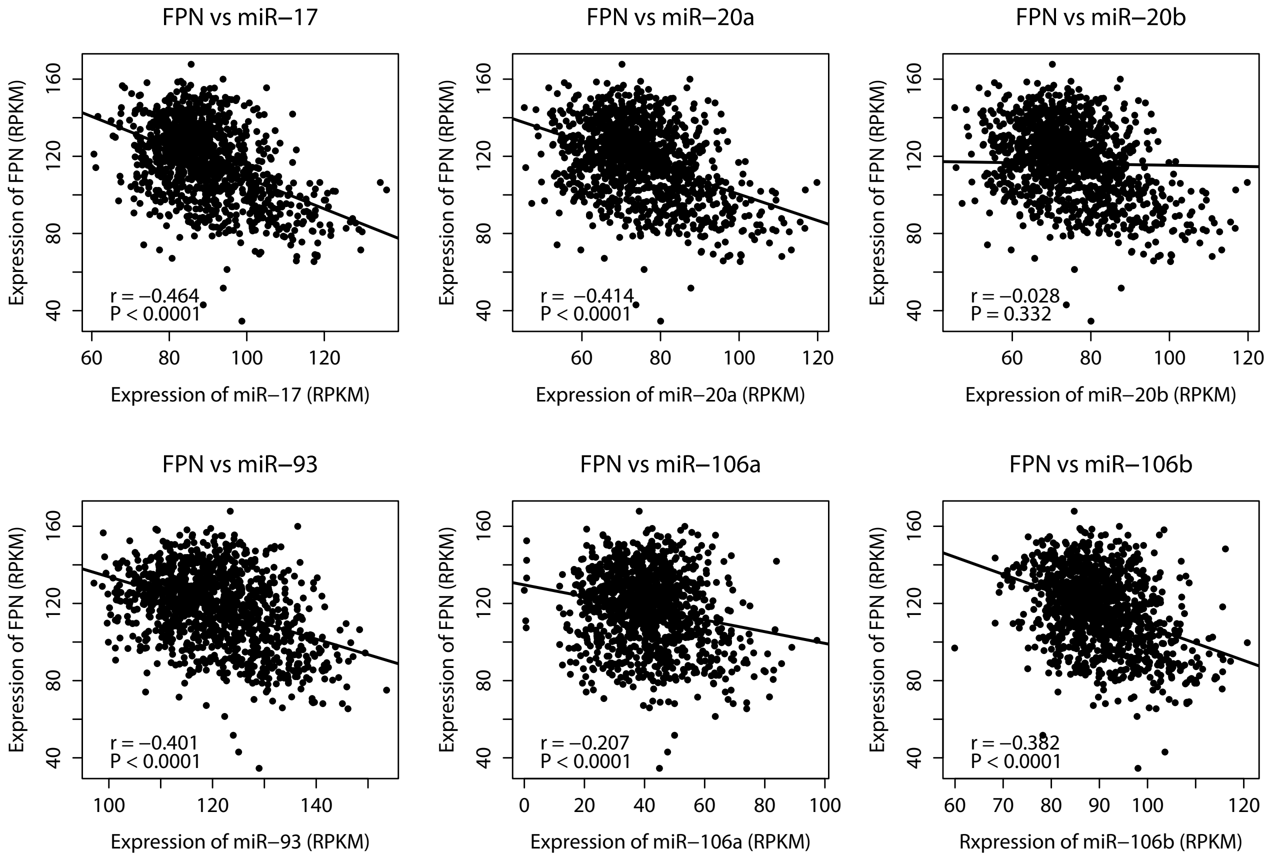

Figure S4. Scatter plots indicating correlation of FPN mRNA levels to the indicated member of the miR-17 seed family within TCGA datasets of (A) hepatocellular carcinoma (LIHC) and (B) breast cancer (BRCA) patients. Pearson’s product moment correlation was used to calculate statistical significance.

Figure S5

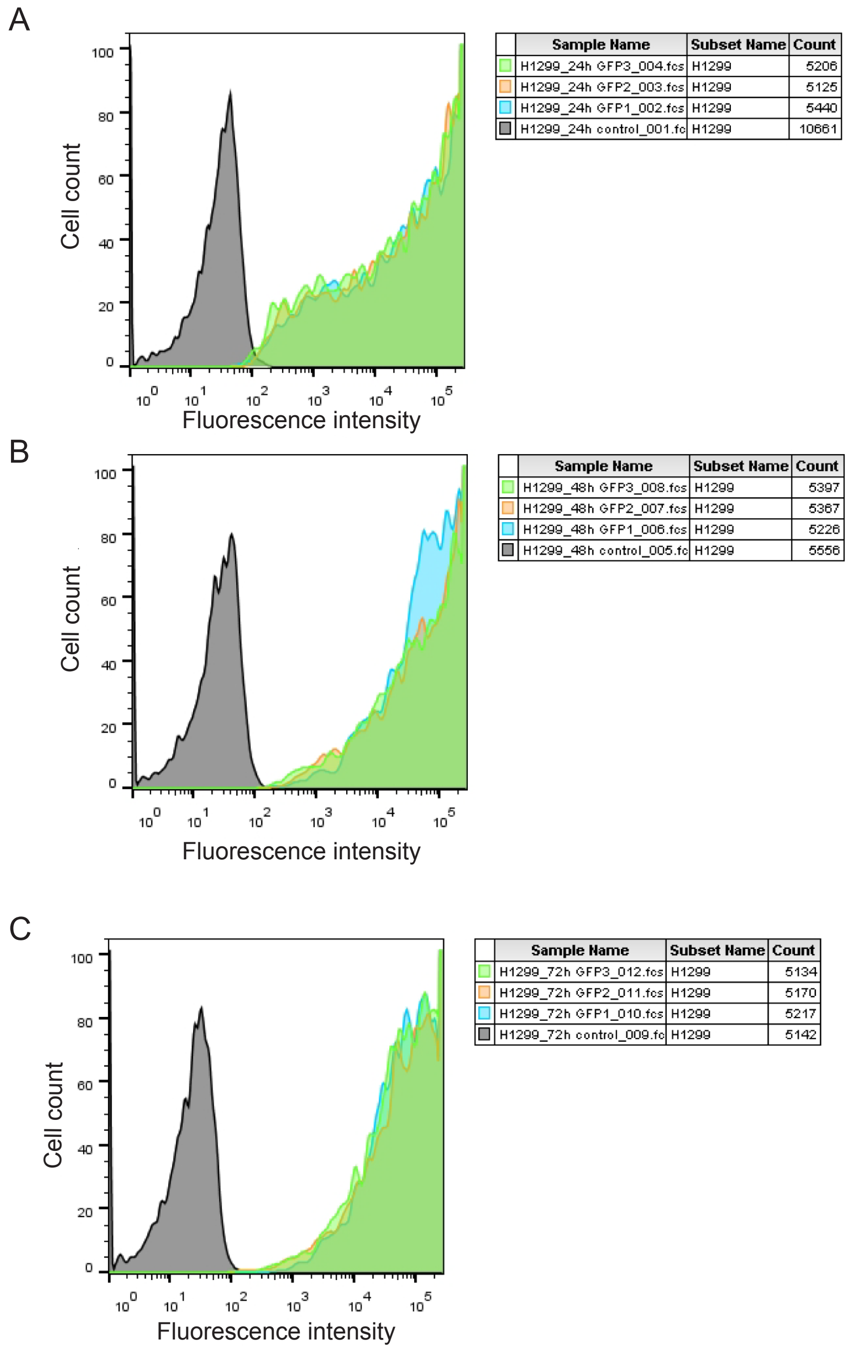

Figure S5. Chromatograms illustrating the transfection efficiency of the FPN expression construct (pcDNA FPN-EGFP) into H1299 cells. Green fluorescence intensity was analyzed by flow cytometry following transient transfection of pcDNA FPN-EGFP into H1299 cells after (A) 24 hrs (B) 48 hrs and (C) 72 hrs.

Figure S6

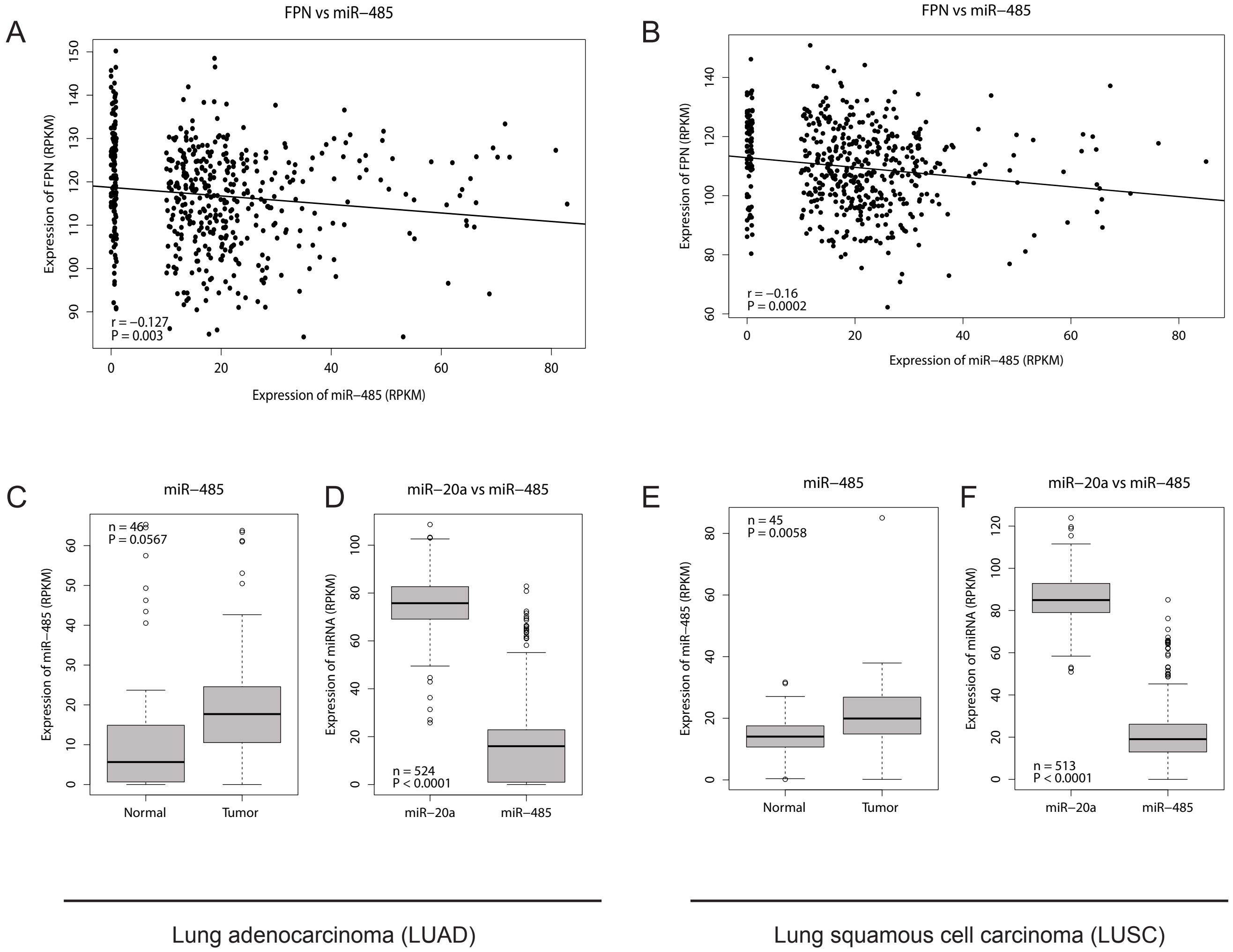

Figure S6. TCGA RNA-sequencing datasets available from Lung adenocarcinoma (LUAD) and Lung squamous cell carcinoma (LUSC) patients were downloaded from the UCSC cancer genomic browser (https://genome-cancer.ucsc.edu). Correlations between miR-485 and FPN mRNA expression levels are represented as scatter plots for (A) LUAD (n = 524, r = -0.127, P = 0.003) and (B) LUSC (n = 513, r = -0.16, P = 0.0002). Pearson's product moment correlation was used to calculate stastistical significance. Boxplots illustrate that in patients with LUAD (C) miR-485 expression is not significantly elevated (n = 46, P = 0.0567) and that (D) miR-485 expression is significantly lower (n = 524, P < 0.0001) compared to miR-20a expression. In patients with LUSC (E) miR-485 is significantly increased compared to normal (n = 45, P = 0.0058) and (F) miR-485 expression is significantly lower (n = 513, P < 0.0001) compare to miR-20a levels. 2-tailed Student's t test was used to calculate statistical significance. RPKM = Reads Per Kilobaseper Million mapped reads.

Figure S7

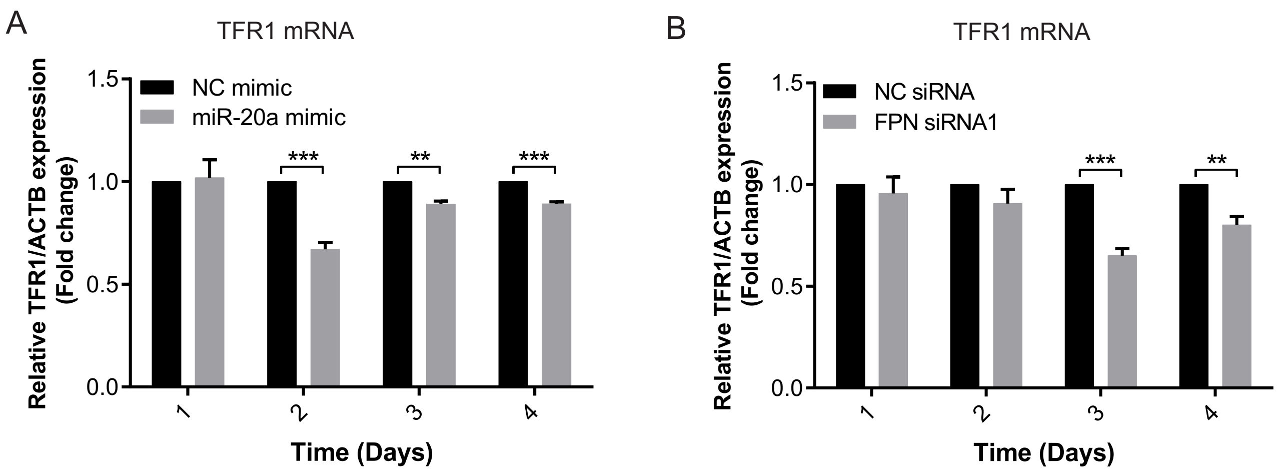

Figure S7. H1650 cells were transfected with 50 nM of (A) miR-20a mimic or (B) FPN siRNA1. After transfection TFR1 mRNA levels were analyzed by quantitative RT-PCR at an interval of 24 hrs for up to 4 days. Experiments were performed in triplicates and repeated at least three times. Data were normalized to ACTB. Data are represented as mean ± SEM, and the values from negative control (NC) mimic were set to 1, ***P* < 0.001, ****P* < 0.001, 2-tailed Student's t test was used to calculate statistical significance.

Figure S8

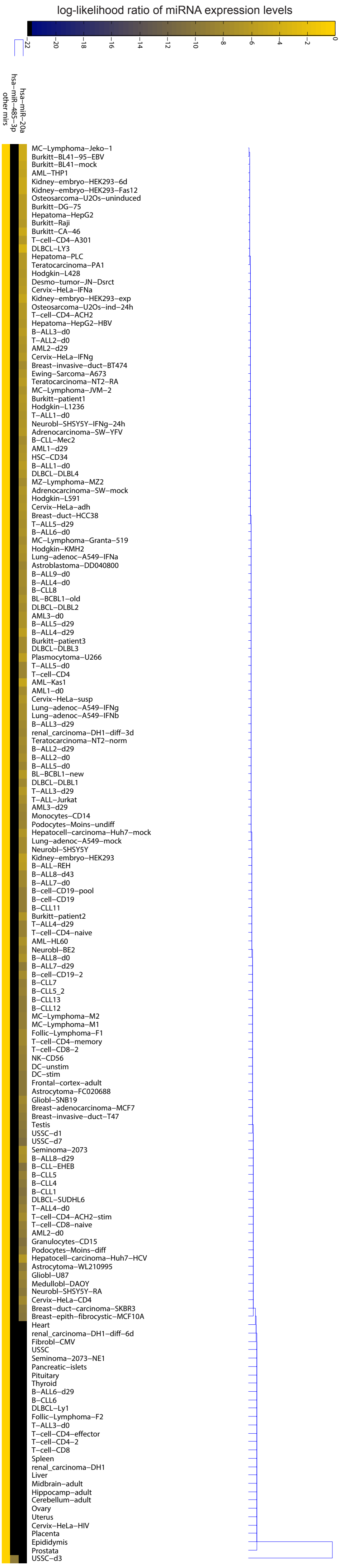

Figure S8. Heatmap illustrating comparative expression levels of miR-20a and miR-485-3p in various human tissues and cell lines.
